# Study on Biogenic Spindle-Shaped Iron-Oxide Nanoparticles by *Pseudostaurosira trainorii* in Field of Laser Desorption/Ionization Applications

**DOI:** 10.3390/ijms231911713

**Published:** 2022-10-03

**Authors:** Piya Roychoudhury, Aleksandra Golubeva, Przemysław Dąbek, Oleksandra Pryshchepa, Gulyaim Sagandykova, Paweł Pomastowski, Michał Gloc, Renata Dobrucka, Krzysztof Kurzydłowski, Bogusław Buszewski, Andrzej Witkowski

**Affiliations:** 1 Institute of Marine and Environmental Sciences, University of Szczecin, Mickiewicza 16a, 70-383 Szczecin, Poland; 2 Centre for Modern Interdisciplinary Technologies, Nicolaus Copernicus University, Wileńska 4, 87-100 Toruń, Poland; 3 Faculty of Materials Science and Engineering, Warsaw University of Technology, Wołoska 141, 02-507 Warsaw, Poland; 4 Department of Industrial Products and Packaging Quality, Institute of Quality Science, Poznań University of Economics and Business, al. Niepodległości 10, 61-875 Poznań, Poland; 5 Faculty of Mechanical Engineering, Białystok University of Technology, Wiejska 45c, 15-351 Białystok, Poland; 6 Department of Environmental Chemistry and Bioanalysis, Faculty of Chemistry, Nicolaus Copernicus University, Gagarina 7, 87-100 Toruń, Poland

**Keywords:** iron-oxide nanoparticles, biogenesis, diatom, LDI-MS, nano-spindle

## Abstract

Nanostructures-assisted laser desorption/ionization mass spectrometry (NALDI-MS) is gaining attention for the analysis of a wide range of molecules. In this present investigation, *Pseudostaurosira trainorii* mediated biosynthesized iron-oxide nanoparticles (IONPs) have been utilized as nanostructures assisting ionization and desorption for laser desorption/ionization mass spectrometry (LDI-MS). The chain forming diatom, *P. trainorii* showed efficiency in the production of IONPs against 0.01 M Fe^+3^ (pH 2) aqueous solution at the intracellular and extracellular level. The whole biomass and external media turned dark orange in color after 3 days of reaction with Fe^3+^ solution. Scanning electron microscopic (SEM) images illustrated that the surface of Fe^3+^ exposed frustules of *P. trainorii* were entirely covered by synthesized nanostructures contrasting with the natural surface ornamentation of control cells. The IONPs loaded frustules also exhibited catalytic properties by decolorizing yellow colored nitrophenol after 3 h of reaction. Transmission electron microscopic (TEM) images confirmed that the produced particles are spindle-shaped with ~50–70 nm length and ~10–30 nm width. The biogenic IONPs were utilized as an inorganic matrix in LDI-MS and showed high sensitivity towards small molecules as glucose, alanine and triacylglycerols at nano- and picomolar level per spot, respectively. The presented biocompatible technique offers new perspectives in nanobiotechnology for the production of spindle-shaped IONPs that can be applied in future for the preparation of NALDI plates.

## 1. Introduction

Iron-oxide nanoparticles (IONPs) are an exceptional and prominent category of metal nanoparticles because of their intrinsic magnetic properties. These magnetic nanomaterials have drawn the attention of biomedical researchers, especially for their utility in hyperthermia-based therapy of cancer due to their heat dissipation ability under an alternating magnetic field [1]. IONPs are also promising candidates for drug delivery [2], catalysis [3], magnetic inks for jet printing [4], killing microorganisms [5], magnetic separation [6], biosensing [7], gene therapy [8], environmental remediation [9] and as contrast agents for magnetic resonance imaging [10] and magnetic fluid hyperthermia [11]. The most used methodologies to produce IONPs are microemulsion, thermal decomposition, co-precipitation, sol–gel [12] and colloidal processing [13]. However, these methods require the usage of hazardous chemicals, such as sodium borohydrate [14], and do not follow the eco-friendly route in IONPs fabrication. Though IONPs are considered completely safe for biological systems, the involvement of such toxic chemicals in synthetic production limits their utilization in medical applications. For this reason, biogenesis has become most popular for the formation of biocompatible IONPs. Some higher plants [15,16], fungi [17,18], bacteria [19,20] have already been exploited as bioreagents for biogenic production of IONPs. Only few reports on algae-based synthesis of IONPs are available. Algae including *Ulva flexuosa* [21], *Padina pavonica* and *Sargassum acinarium* [22], *Gracilaria edulis* [23], *Kappaphycus alvarezii* [24], *Turbinaria decurrens* [25] showed efficiency in production of IONPs against ferric chloride solution. However, no reports are available regarding diatom mediated production of IONPs. Diatom inspired approaches become most successful in the construction of silica conjugated metal nanoparticles. Doping of metal particles on a layer of silica is sometimes essential to make the particles more stable, harder, and more effective for catalysis [26]. Certain metal nanoparticles, namely gold, silver, platinum, titanium, germanium, palladium, have already been modified by using the natural siliceous shells of *Amphora copulata* [27], *Halamphora subturgida* [28], *Coscinodiscus wailesii* [29], *Thalassiosira weissflogii* [30], *Pinnularia* sp. [31], *P. trainorii* [32], respectively. In this study, we have reported the bio-protocol for construction of IONPs decorated frustules of *P. trainorii* for the first time.

p-Nitrophenol is considered as one of the most highly toxic compounds for the environment as it consists of nitro group in its aromatic ring structure. p-Nitrophenol is commonly used in pharmaceuticals, fungicides, insecticides, and dye industries. This pollutant from contaminated industrial wastes is continuously entering the ecosystem and induces significant health risks. Presently, scientists are trying to degrade p-nitrophenol by exploiting the catalytic activity of various nanoparticles. It has been reported that iron nanoparticles synthesized by *Jatropha* [33] and *Artocarpus heterophyllus* [34] leaf extracts showed potential catalytic activity to degrade nitrophenol. It was also reported that iron oxides are capable of degrading nitrophenol in the presence of oxalate [35]. Fe^3+^/iron oxide/SiO_2_ xerogel also showed catalytic effect in nitrophenol reduction by photo-fenton reaction [36]. Therefore, it can be said that biogenic IONPs doped frustules of *P*. *trainorii* will be an effective photocatalytic agent for nitrophenol degradation.

Nanoparticles with a wide center portion and having both tapering ends in the size ranges of 1–100 nm in any dimension are known as nanospindle, a prominent category of various nanoforms. Among the various shapes, rod/spindle-shaped particles are gaining attention because they are more effective in photothermal therapy. Increased accumulation of spindle-shaped nanoparticles at the tumor site than spherical particles was also reported [37]. Chemically synthesized rod-shaped IONPs showed more potential effect in magnetic hyperthermia than polyhedral shape [37]. Few authors reported bioreagents mediated production of rod/spindle-shaped IONPs. Brayner et al. 2009 [38] reported that rod-shaped akaganeite (β-FeOOH) nanoparticles production at the intracellular level is possible by cyanobacteria, *Anabaena* sp. and *Calothrix* sp. and green alga, *Klebsormidium* sp. at room temperature. Spindle-shaped zero valent nanoiron was synthesized by *Arthrospira platensis* [39] and *Leptolyngbya valderiana* [40]. No report is available regarding diatom assisted biogenic production of spindle-shaped IONPs.

Matrix-assisted laser desorption/ionization (MALDI) has become an important tool for the determination of molecular mass of macromolecules such as proteins. In contrast, MALDI has limitations for applications below *m*/*z* 500 due to suppression effects and chemical background interfering with low molecular weight analytes [41]. In addition, various nanomaterials were applied as inorganic matrix for analysis of low molecular weight compounds in LDI-MS. Gold, silver, zinc oxide, platinum nanoparticles have been used as matrix and showed significant sensitivity for determination of molar mass of carbohydrate [42], triglycerides [43], polyethylene glycol [44], saccharides [45], respectively. IONPs have been used in MALDI instead of matrix for analysis of glycan and peptides [46]. Citric acid capped IONPs have been recognized as an effective matrix for polymers [47]. Dihydrobenzoic acid modified IONPs has been used as matrix for the determination of diverse structures of small molecules [48]. Therefore, it was hypothesized that IONPs synthesized in the proposed, cost-effective way, may serve as nanomaterials assisting with ionization and desorption in LDI-MS analysis of low molecular weight analytes.

In the present investigation, spindle-shaped IONPs have been synthesized using diatom-strain, *P. trainorii* (BA170) E.A. Morales (2001) [49] procured from the Culture Collection of Baltic Algae, Institute of Oceanography, University of Gdańsk, Poland with further detailed characterization using analytical techniques followed by an evaluation of LDI-MS efficiency of IONPs as a nanomaterial for the preparation of NALDI plates.

## 2. Results

### 2.1. Diatom Mediated Biofabrication of IONPs

**Figure 1:**
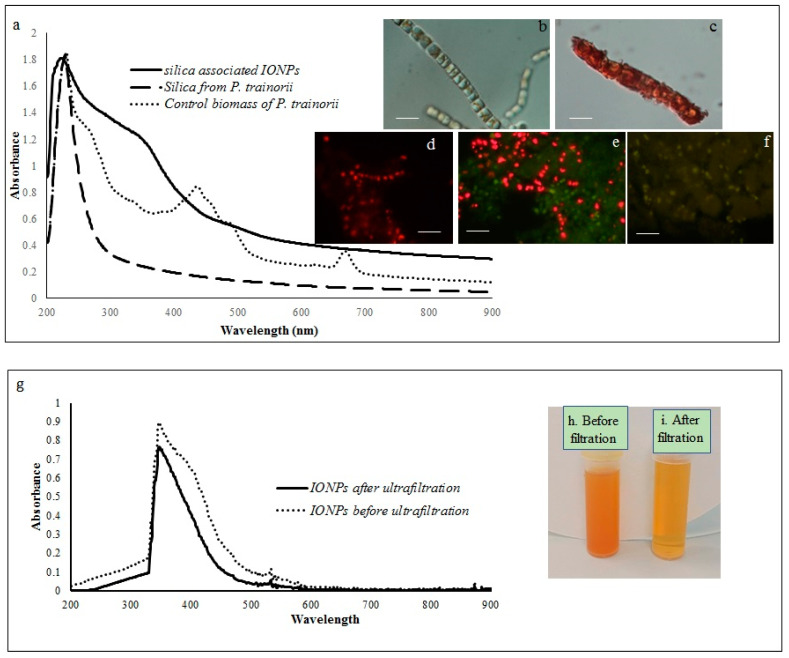
Showing UV-Vis spectroscopy of control biomass, cleaned frustules and IONP-loaded frustules (**a**). LM images of *P. trainorii* before (**b**) and after (**c**) treatment with Fe^3+^ solution [scale bar 1 μm]. Fluorescent images control (**d**) and Fe^3+^ exposed *P. trainorii* taken at 24 h (**e**), 72 h (**f**) of reaction [scale bar 1 μm]. UV-Vis spectroscopy of extracellular IONPs before and after ultrafiltration (**g**). Extracellular IONPs suspension before (**h**) and after (**i**) ultrafiltration.

The reduction of Fe^3+^ ions and subsequent production of biogenic IONPs at the intracellular and extracellular level was initially detected by observing color changes in biomass and experimental media. The whole biomass of *P. trainorii* exhibited a time-dependent color change during 3 days of the incubation period with 0.01 M (pH 2) Fe^3+^ solution. The yellowish-green cells (Figure 1b) of *P. trainorii* started to turn orangish in color after 24 h of reaction. However, all cells of *P. trainorii* turned dark orange in color after 3 days of reaction (Figure 1c). Not only that, but the external media also turned dark orange in color (Figure 1h,i). After 3 days of reaction, no further color change in cells and in experimental media was observed. It was noted that during the development of orange color, *P. trainorii* lost all its chlorophyll and carotenoids contents (Figure 1c), which was also confirmed by fluorescent microscopy (Figure 1d–f) and UV-Vis spectroscopy (Figure 1a). Under the fluorescent microscope at 450–490 nm excitation the control cells of *P. trainorii* showed red fluorescence of chlorophyll (Figure 1d). However, after 24 h of Fe^3+^ exposure maximum cells of *P. trainorii* showed green fluorescence property (Figure 1e) as well as after 3 days of reaction all cells only emitted green fluorescence (Figure 1f). The metal stress related morphological changes, e.g., the high rate of cell division (Figure 1e) and loss of cellular integrity (Figure 1f) were also documented in Fe^3+^ treated *P. trainorii* which signified iron toxicity.

### 2.2. SEM Analysis of Fe^3+^ Treated P. trainorii with EDAX and Elemental Mapping

**Figure 2:**
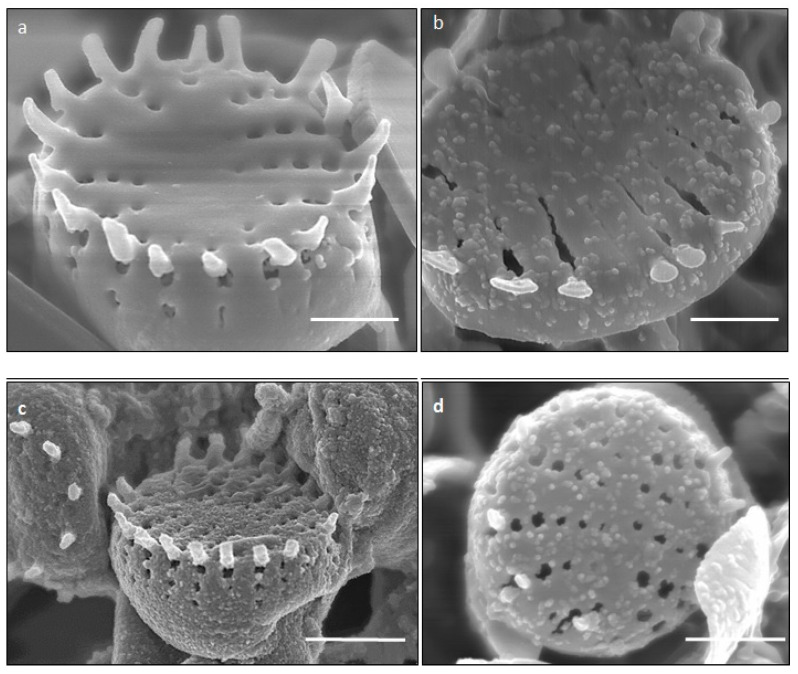
SEM images showing surface morphology of control (**a**) and IONPs loaded (**b**–**d**) frustules *P. trainorii* [scale bar 1 μm].

**Figure 3:**
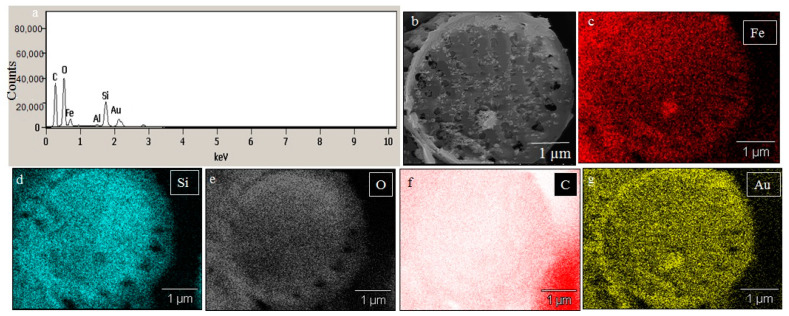
EDAX spectra of IONPs loaded frustules (**a**). Elemental mapping of IONPs loaded single frustules confirmed presence of iron, silicon and oxygen all over the frustules (**b**–**g**).

SEM images illustrated the surface decoration of siliceous frustules of *P. trainorii* before (Figure 2a) and after treatment with Fe^3+^ solution (Figure 2b–d). SEM micrographs revealed the biosynthesis of nanostructures and their deposition on the surface of Fe^3+^ treated *P. trainorii* (Figure 2b–d). Surfaces of Fe^3+^ exposed frustules were entirely covered by synthesized nanostructures contrasting natural surface ornamentation of control cells. Elemental mapping (Figure 3b–g) as well as EDAX (Figure 3a) study of nanoparticles loaded cells confirmed the presence of silicon, oxygen, and iron all over the frustules. A strong signal of gold (Au) was also observed as Au was used as coating material for SEM study. Elemental mapping of a single frustule loaded with IONPs illustrated the distributional pattern of the elements, namely silicon, oxygen, and iron (Figure 3b–g).

### 2.3. UV-Vis and Fourier Transform Infrared Spectroscopy

A comparative spectral analysis among water extracts of control biomass, cleaned frustules and IONPs loaded frustules of *P. trainorii* have been performed. In UV-Vis spectroscopy, water extracts of the control biomass of *P. trainorii* showed three distinct peaks at 270, 450, and 663 nm (Figure 1a). These three consecutive peaks at 270, 450, 663 nm revealed the presence of silica, carotenoids and chlorophyll, respectively, in untreated cells. The H_2_O_2_ washed frustules’ extract of *P. trainorii* showed maximum absorbance at 270 nm and confirmed the presence of only silica particles as pigment content was below the detectable limit (Figure 1a). The Fe^3+^ exposed orange colored biomass’ extract of *P*. *trainorii* showed two characteristic peaks at ~270 and ~350 nm, respectively (Figure 1a). The extracellular brown suspension also showed distinct peak at ~350 nm before and after ultrafiltration (Figure 1g).

**Figure 4:**
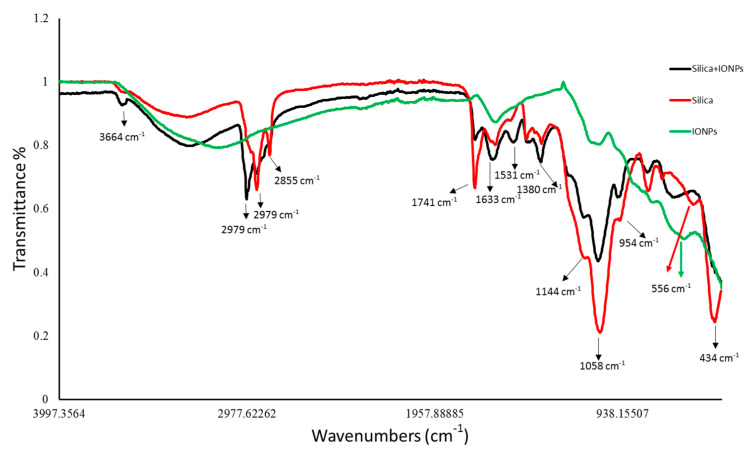
FTIR peaks revealed the functional groups present surrounding the surfaces of control silica, silica loaded particles and extracellular IONPs.

FTIR spectra revealed the chemical nature of control and IONPs loaded frustules of *P. trainorii* as well as extracellular IONPs as shown in Figure 4. During the measurement of control silica and silica doped IONPs, peaks appeared at 3664, 2979, 2924, 2855, 1741, 1633, 1531, 1380, 1144, 1058, 954, 434 cm^−1^. These peaks revealed the presence of O–H, C–H, C=O, N–H, Si–O–Si and Si–O functional groups on the surface of frustules as well as frustules loaded with synthesized IONPs. However, a distinct peak at 556 cm^−1^ has been observed only in IR spectra of silica loaded IONPs and externally produced IONPs which has been identified as characteristic peak of Fe-O.

### 2.4. ICP-MS, Zeta Potential and X-ray Diffraction

**Figure 5:**
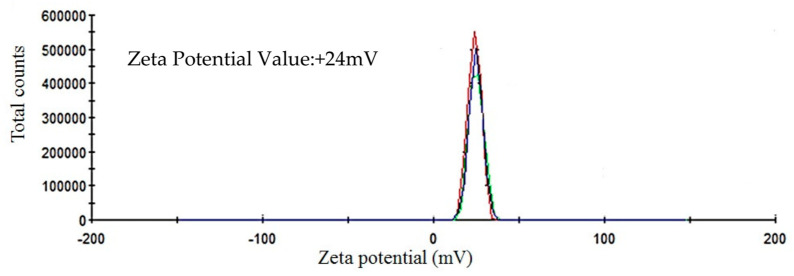
Showing zeta potential value of extracellular IONPs.

**Figure 6:**
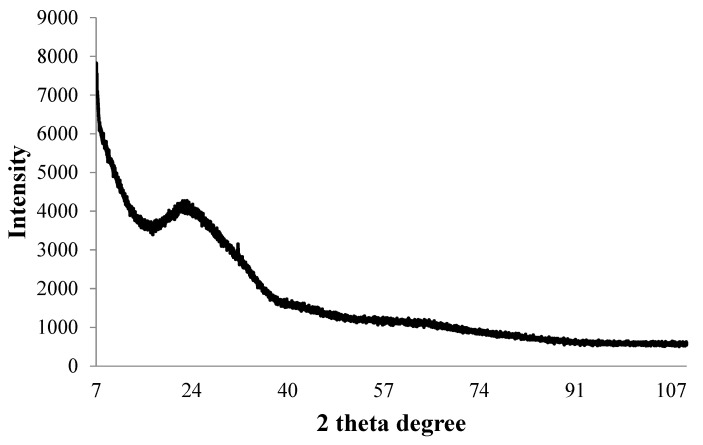
X-ray diffraction pattern of synthesized IONPs by *P. trainorii*.

The ICP-MS study confirmed that the concentration of ultrafiltered extracellular IONPs suspension was 152 mgL^−1^. Zeta potential values of IONPs were measured as +24.6 mV (Figure 5). It stated that produced nanoparticles had positively charged surface. The XRD spectra did not show any specific peak for externally synthesized IONPs (Figure 6) and revealed that synthesized IONPs were amorphous in nature.

### 2.5. TEM and EDAX

**Figure 7:**
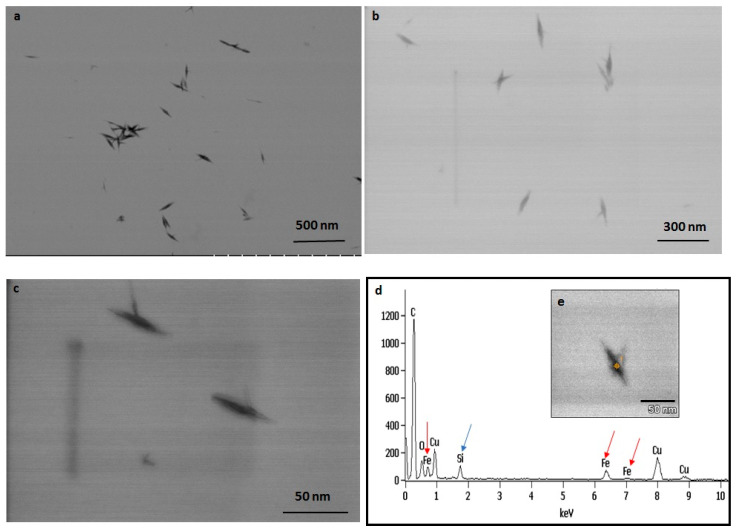
TEM images of extracted IONPs from particle loaded frustules captured in various magnification (**a**–**c**). EDAX study of single particle confirming presence of iron, silica and oxygen (**d**). TEM image of single spindle shaped IONP (**e**).

**Figure 8:**
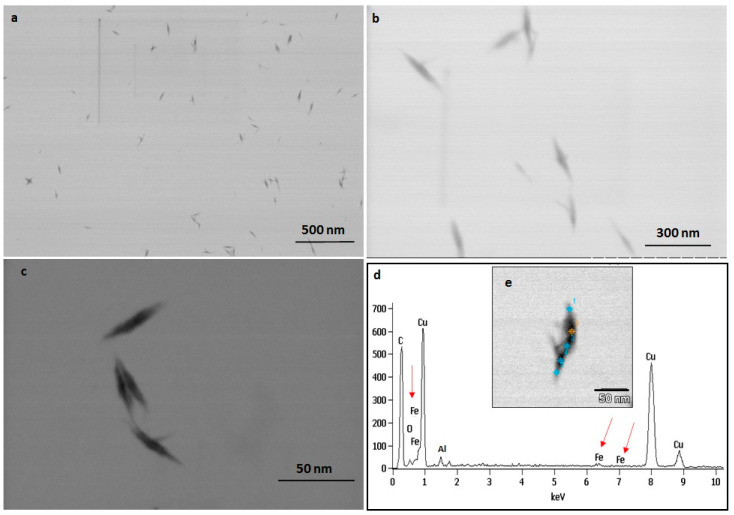
TEM images of extracellular IONPs captured in various magnification (**a**–**c**). EDAX study of single particle confirming presence of iron, oxygen (**d**). TEM image of single extracellular IONP (**e**).

The TEM observations represent a clear picture regarding the shape and size of IONPs produced by the experimental taxa (Figure 7 and Figure 8). The TEM study confirmed that all synthesized particles (extracted and extracellular) are spindle-shaped with almost the same size. The average length of the nano-spindle was in the range of 50–70 nm with the width of 10–30 nm range.

The EDX spectra of extracted and extracellular IONPs are shown in Figure 7d and Figure 8d, respectively. The EDX spectra of extracted nanostructures from NP loaded frustules showed three distinct peaks, which corresponds to the signals of silica, oxygen, and iron, respectively. The same signals were observed from seven different spots of a single particle. However, EDX spectra of extracellular IONPs showed only two strong signals of iron and oxygen.

### 2.6. 4-Nitrophenol Degradation by IONPs Decorated Frustules

**Figure 9:**
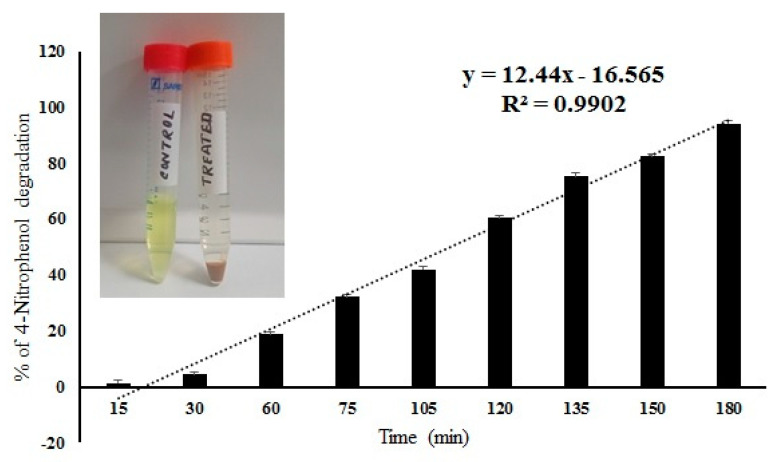
Percentage of 4-nitrophenol degradation by IONPs doped frustules of *P. trainorii* and experimental solution of 4-nitrophenol before and after treatment with IONPs loaded frustules of *P. trainorii*.

The IONPS decorated frustules of *P. trainorii* showed positive responses in degradation of 4-nitrophenol under light. The degradation of 4-Nitrophenol after exposure to IONPs loaded frustules was preliminarily confirmed by the color change of nitrophenol. During the reaction, gradually, the yellow color of nitrophenol faded away with time. After 3 h of reaction, the solution became completely colorless. Spectroscopic data showed a sharp decrease in absorbance with an increase in reaction time, which revealed the degradation of 4-nitrophenol (Figure 9). After 180 min of reaction, 94% ± SE 0.157 of 4-nitrophenol degradation was confirmed by spectroscopic analysis (Figure 9). The control set of 4-nitrophenol without IONPs loaded frustules did not show any significant change (Figure 9).

### 2.7. LDI-Mass Spectrometry

**Figure 10:**
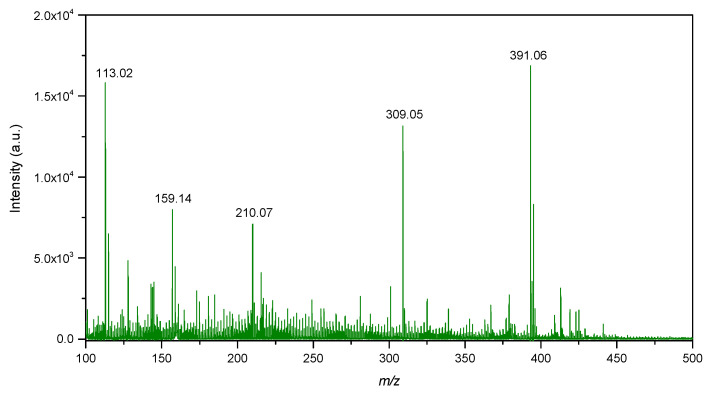
LDI-MS spectra of IONPs in the low mass region.

**Figure 11:**
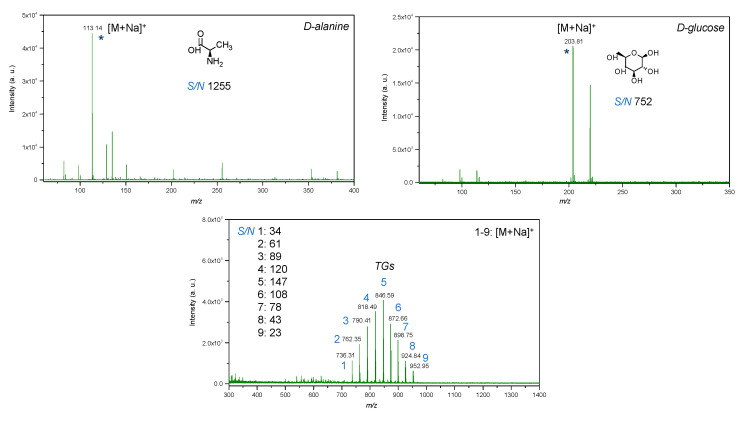
LDI-MS spectra of low molecular weight compounds (glucose, alanine, mixture of triacylglycerols) with utilization of IONPs as an inorganic matrix, where: 1—14:0-13:0-14:0 TG (25 μg/mL); 2—14:0-15:1-14:0 TG (50 μg/mL); 3—14:0-17:1-14:0 TG (75 μg/mL); 4—16:0-15:1-16:0 TG (100 μg/mL); 5—16:0-17:1-16:0 TG (125 μg/mL); 6—16:0-19:2-16:0 TG (100 μg/mL); 7—18:1-17:1-18:1 TG (75 μg/mL); 8—18:1-19:2-18:1 TG (50 μg/mL); 9—18:1-21:2-18:1 TG (25 μg/mL); * is molecular ion.

The LDI-MS spectra of biogenic IONPs utilized as nanomaterial assisting ionization and desorption is presented in Figure 10. IONPs utilized for LDI-MS analysis showed sensitivity towards low molecular weight analytes such as glucose, alanine and triacylglycerols (Figure 11).

## 3. Discussion

It is well known that pure iron or IONP has a distinct color that varies from yellow to dark brown and further to complete black depending upon the size, shape and concentration of the particles [39]. It is reported by many authors that faint yellowish FeCl_3_ solution turned dark orange in color during interaction with some reducing agents due to the production of pure iron [39]/IONPs [50]. Likewise, in this study, the occurrence of orange color in the green biomass of *P. trainorii* as well as in the medium initially confirmed the development of iron associated nanostructures at intracellular and extracellular level. However, the color change could not reveal the production of IONPs which was later confirmed by EDAX, XRD and LDI-Mass spectrometry. A preliminary color change was observed after 12 h of exposure; therefore, it can be said that production of IONPs by *P. trainorii* have started after 12 h of reaction and the reduction procedure has been completed after 3 days as no further color change was observed. Extracellular nanoparticle production is more acceptable since the extraction steps, e.g., ultrasonication and vortexing, are not required. On the other hand, cell-associated nanoparticles are needed to extract using some additional nano-capping agents, such as sodium citrate [51], and cetyltrimethyl ammonium bromide (CTAB) [52] to control their stability and to keep away from aggregation. During such extraction, some cellular fragments can be associated with the nanoparticles, whereas extracellular production is comparatively pure. Therefore, it can be inferred that extracellular fabrication of IONPs by *P. trainorii* is a timesaving, biocompatible and cost-effective technique.

Diatoms are natural, attractive bioreagents for continuous production of amorphous nanosilica within silica deposition vesicle [53]. These silica nanoparticles are responsible for the generation of a mesoporous, siliceous outer covering, known as frustules. Frustules are promising resources for nanobiotechnology because of their easy availability, biocompatibility, porous structure and high surface area. The frustule’s morphology is species-specific and the initial key for species identification. Not only in taxonomic identification, diatomaceous biosilica has also been exploited as a catalyst [54], in optical devices and biosensors [55] and as a drug delivery vehicle [56] because of its unique optoelectrical property. The pure form of these amorphous nanostructures can easily be fabricated in laboratory conditions by culturing diatoms. In this study, *P. trainorii* has been cultured in laboratory conditions without any contamination and further used as reducing agents for production of iron doped siliceous frustules. Chemically synthesized iron doped silica nanoparticles have already been reported as catalyst [57] and potential agents for magnetic resonance imaging [58]. Moreover, frustules are widely used in material engineering for fabrication of three-dimensional metal doped nanosilica because silica provides extra stability to metal nanoparticles as well as intrinsic luminescent property. However, physical and chemical process to fabricate silica conjugated metal particles involve high temperature/pressure and hazardous chemicals, respectively. In this study, the development of silica doped IONPs production was possible at room temperature without involving any toxic chemicals, which was confirmed by SEM study. Additionally, the IONP doped frustules also showed 4-nitrophenol degradation efficiency. The decolorization of 4-nitrophenol possibly happened due to its reduction into aminophenol as mentioned by Tang et al. (2015) [57] while utilizing zero-valent iron particles immobilized on mesoporous silica in reductive degradation of aqueous p-nitrophenol.

In UV-Vis spectroscopy of extracted IONPs from NP loaded frustules, the appearance of two peaks at ~270 and ~350 nm confirmed the presence of both silica and IONPs in the suspension; it is well known that pure silica shows a distinct peak in spectroscopy at 230–290 nm [51]. Extracellular IONPs showed only single peak at 350 nm, confirming no contamination of nanosilica. Chemically synthesized IONPs by the co-precipitation method also showed maximum absorbance at 340 nm in UV-Vis spectroscopy [59]. Biogenically synthesized iron particles by *Ageratum conyzoides* [60] and *Terminalia chebula* [61] leaf showed surface plasmon resonance at 390 nm and 327 nm, respectively. The composite IONPs (Fe_3_O_4_/α-Fe_2_O_3_) fabricated by pulp of *Syzygium cumini* exhibited maximum absorbance at 350 nm [62]. Therefore, it can be concluded intra- and extracellular production of IONPs by *P. trainorii*. FTIR measurement was performed for identification of the potential surface molecular constituents responsible for reduction of Fe^3+^. In IR spectrum of extracellular IONPs and IONPs doped frustules, the appeared peak at 556 cm^−1^ confirmed the presence of Fe-O group as the same was observed by Yew et al. 2016 [24] while synthesizing IONPs using seaweeds *Kappaphycus alvarezii*. The characteristic IR peaks for silica were observed in both control and particles loaded frustules at 434, 954, 1058, 1144 cm^−1^ corresponds to bending vibration and asymmetrical stretching of Si-O-Si [63]. The peaks at 1380, 1531, 1633 cm^−1^ have been observed for strong bending vibrations of N–H functional groups [64]. The peak at 1741 cm^−1^ indicated C=O stretching [64,65,66]. The signals at 2855, 2924, 2979 cm^−1^ were referred as C–H stretching [64,67]. The signal at 3664 cm^−1^ was for O–H stretching [64]. Therefore, it can be said that the identified functional groups, N–H, C=O, C–H provided efficient stabilization of fabricated IONPs.

The stability of IONPs was also confirmed by high positive (+24.6 mV) zeta potential value. High positive or negative surface charge (±20 mV) reveals particles stability as the particles with high zeta potential value repel each other, prevent aggregation and precipitation. The recorded XRD spectra for IONPs is very similar with the XRD data obtained by other authors while producing amorphous IONPs chemically [68] or biogenically [40]. So, it can be said that synthesized particles are amorphous in nature.

The TEM study revealed the advantage of using *P. trainorii* as a reducing agent in the production of IONPs. This bioreagent produced only spindle-shaped IONPs against the 10 mM FeCl_3_ (pH 2). It is difficult in biogenesis to produce similar shapes, because all the reducing agents, e.g., pigments, proteins, polysaccharides, etc., work together against metal stress. Therefore, it is common to synthesize particles with variable shapes and sizes. However, in this method, it was possible to synthesize only spindle-shaped particles without any trace of other shapes. EDAX study of extracellular particles confirmed the production of IONPs as two strong signals from Fe and O have been detected. On the other hand, sonicated out particles from frustules (loaded with particles) showed an additional signal of silica in EDAX. Therefore, it can be said that extracellular particles have been characterized by higher purity and lack of silica contamination.

LDI-MS spectra showed intense signals in the low mass region at *m*/*z* 113.02, 159.14, 210.07, 309.05 and 391.06 (Figure 10). The signal at *m*/*z* 113.02 probably corresponds to FeO as adduct with 1 molecule of water and sodium [M+H_2_O+Na]^+^. The signal at *m*/*z* 391.06 probably corresponds to Fe_4_O_5_ as adduct with 1 molecule of water and 3 atoms of sodium [M+H_2_O+3Na]^+^. On the other hand, isotopic distribution of the signals was not observed to be similar as for iron oxide compounds. Therefore, mentioned signals could also correspond to organic compounds originating from the synthesis of IONPs.

LDI-MS spectra of standards of low molecular weight compounds with utilization of IONPs as matrix showed intense signals in low mass region. All standards such as glucose, amino acid and triacylglycerols were detected as adducts with sodium [M+Na]^+^ (Figure 11). It was expected since trace amounts of sodium may be present in water used for the synthesis and in addition, as compared to silver and gold nanostructures, iron oxides do not tend to form adducts with analytes. Notably, the LDI-MS spectra of glucose demonstrated less rich chemical background in low mass region as compared to alanine, that probably can be explained by differences in distribution of standard compounds on the surface of IONPs. Since IONPs were deposited by pipetting onto the plate, which was followed by drying on air, the coffee ring effect [69] could affect the distribution of analytes on the surface of IONPs and should be further studied for their further applications in LDI-MS. The deposition method of the nanomaterial onto NALDI plate affects its distribution and therefore, LDI spectra, further studies are necessary for evaluation of applicability of IONPs for preparation of NALDI plates with utilization of presented approach. For example, techniques such as magnetron sputtering and vapor deposition allow for a higher degree of control rather than wet and dry chemical methods. Pre-mixing prior to deposition provided insignificant difference in intensity for alanine, and in contrast, intensity for glucose was observed to be three times higher as compared to dried droplet method. This probably can be caused by differences in chemical structure of compounds and thus, interactions between analytes and IONPs.

In addition, it is important to note that chemical background of IONPs did not interfere with molecular ions of low molecular weight compounds that were detected at nanomolar level (per spot).

Triacylglycerols were detected at picomolar detection level (per spot), which shows high sensitivity of LDI-MS technique with utilization of IONPs as inorganic matrix. As compared to the previous work with utilization of silver nanostructures [70], it was not necessary to adjust parameters of global attenuator and laser parameter set as well as the value of detector gain to obtain intense molecular ions for triacylglycerols. Furthermore, TG 9 at *m*/*z* 952.95 was not detected in case of utilization of silver nanostructures as matrix. Such a difference can probably be explained by differences in catalytic properties since silver is possessed as highly active catalyst as compared to iron, which may lead to enhanced fragmentation of lipids and absence of the molecular ion, thus complicating identification. Furthermore, pre-mixing of IONPs and TGs prior to deposition onto the LDI plate provided less intense molecular ions as compared to dried droplet technique.

Values of signal-to-noise ratio were high for all standard compounds, which shows that the limit of detection of the technique with utilization of IONPs as nanostructures assisting ionization and desorption processes is potentially significantly lower than utilized concentrations (Figure 11).

Diatoms are excellent bioreagents for biosynthesis of various nanoparticles because of their high growth rate and high metal uptake capacity. These microscopic, silica nanofactories have already been exploited for biogenic production of various nanoparticles, namely gold [71], silver [51]. Since the production of nanoparticles is common in the metal stress response of diatoms against particular reducing environment. Here, *P. trainorii* showed pigment loss against Fe^3+^ stress as well as spindle-shaped IONPs production. The gradual disappearance of red fluorescence in Fe^3+^ treated *P. trainorii* confirmed loss of chlorophyll. The loss of chlorophyll and carotenoids in Fe^3+^ treated *P. trainorii* was also recorded by observing no peak at 663 and 450 nm in UV-Vis spectroscopy, respectively. The appearance of only green fluorescence after 3 days of reaction confirmed cell death as silica shows intrinsic green luminescent property under blue light region. A fragment of IONPs associated frustules would be beneficial for optical imaging guided cancer therapy.

## 4. Materials and Methods

### 4.1. Chemicals

Thiamine hydrochloride (99%, MW 337.27), biotin (>99%, MW 244.31), vitamin B12 (>98%, MW 1355.37), and α-Cyano-4-hydroxycinnamic acid (>99%, MW 189.17), 4-Nitrophenol (>99%, MW 139.11) were supplied by Sigma-Aldrich (St. Louis, MO, USA). Hydrogen peroxide (30%, MW 34.01), sodium nitrate (>99%, MW 84.99), sodium dihydrogen phosphate monohydrate (>99%, MW 137.99), sodium molybdate dihydrate (>99%, MW 241.95), manganese (II) chloride tetrahydrate (>99%, MW 197.91), and cobalt (II) chloride hexahydrate (>99%, DW 237.93) were obtained from Chempur^®^ (Piekary Śląskie, Poland). Zinc sulfate heptahydrate (>99%, MW 287.54), iron (III) chloride hexahydrate (>99%, MW 270.32), EDTA disodium dihydrate (>99%, MW 372.24), and copper (II) sulfate pentahydrate (>99%, MW 249.68) were purchased from Scharlab (Barcelona, Spain). Sodium metasilicate nonahydrate (44–47.5% total solids, MW 284.19) were supplied by Acros Organics, ThermoFisher Scientific (Waltham, MA, USA). Deionized water was obtained by using a Milli-Q^®^ purification system (Millipore Co., Bedford, MA, USA). Standards of D-glucose (>99%), D-alanine (>99%) and cesium triiodide (>99%) were purchased from Sigma-Aldrich (Steinheim, Germany). Standard mixture of triacylglycerols (TG internal standard, Ultimate SPLASH^TM^) was purchased from Avanti Polar Lipids (Alabaster, AL, USA).

### 4.2. Diatom Mediated Biosynthesis of IONPs

#### 4.2.1. Cultivation of Diatom

The pure culture of the selected diatom strain, *P. trainorii* BA170 (collected from Gulf of Gdańsk, Baltic Sea, Poland) was procured from Diatom Culture Collection, University of Szczecin, Poland. The culture was maintained using standard 7 ppt Guillard’s artificial seawater f/2 medium [72] (1 L of medium contains 880 µM NaNO_3_, 36 µM NaH_2_PO_4_ H_2_O, 106 µM Na_2_SiO_3_ 9H_2_O, trace metal: 0.08 µM ZnSO_4_ 7H_2_O, 0.9 µM MnSO_4_ H_2_O, 0.03 µM Na_2_MoO_4_ 2H_2_O, 0.05 µM CoCl_2_ 6H_2_O, 0.04 µM CuCl_2_ 2H_2_O, 11.7 µM FeCl_3_ 6H_2_O, 11.7 µM Na_2_EDTA 2H_2_O, vitamin B12, biotin and thiamine) at 20 °C under a 12:12 day:night cycle, illuminated with 100 μmol photons m^−2^ s^−1^ of white light. The strain was cultivated for 14 days to obtain healthy growing biomass from exponential growth phase.

#### 4.2.2. Biofabrication of Spindle-Shaped IONPs

The whole biomass of diatom strain *P. trainorii* was used as reducing agent for the production of biogenic spindle-shaped IONPs. The healthy cells of *P. trainorii* were centrifuged at 6000 rpm for 10 min, after that washed with double distilled water (ddH_2_O) for three times to remove excess salts from medium and recollected followed by an additional step of centrifugation at 6000 rpm for 10 min. The thoroughly washed biomass (300 mg FW) was exposed to 400 mL, 0.01 M Fe^3+^ solution with pH 2. The whole experiment was maintained in dark at room temperature for 3 days.

#### 4.2.3. Purification of Diatom Based Biosynthesized IONPs

After 3 days of reaction, the resultant golden yellow colored biomass was collected by centrifugation at 8000 rpm for 20 min. The harvested biomass was rinsed 3–4 times using ddH_2_O and stored at 4 °C for further characterizations. The orange colored external nanosuspension was also collected separately. The produced IONPs at extracellular level were purified by ultrafiltration using Amicon^®^ Ultra Ultracel-30 regenerated cellulose membrane centrifugal filters with NMWL 30 kDa. The ultrafiltration was performed by centrifugation of 15 mL of external nanosuspension at 10,000 rpm for 15 min followed by a thorough wash (3 times) of accumulated particles using deionized water. After washing, the particles were dispersed within water by sonication in ultrasonic water bath for 20 min followed by a 2 min of vortexing at 2000 rpm using Vortex-Genie^®^ 2 (Scientific Industries, Inc., New York, NY, USA) The consecutive steps of sonication and vortexing were repeated three times. After that, the pH of water dispersed nanoparticles was measured by FiveEasy Plus pH meter (Mettler Toledo, Columbus, OH, USA). The cleaned, well dispersed IONPs were stored at 4 °C for further experiments.

### 4.3. Microscopic Analysis of Fe^3+^ Treated P. trainorii with EDAX and Elemental Mapping

Associated morphological differences in *P. trainorii* before and after 3 days of reaction with Fe^3+^ solution were documented by producing light microscopic images with ZEISS Axioscope A1 (Jena, Germany) at 400× magnification. The changes of fluorescent properties in NPs loaded cells were observed by an Axioscope A1 Zeiss fluorescence micro-scope (excitation: 450–490 nm and emission: 515 nm). To record surface topography of control and Fe^3+^ treated *P. trainorii*, the SEM images were captured using a Hitachi SU8020 (Hitachi, Tokyo, Japan). For SEM study, the 40 µL of samples were dried overnight on Nuclepore™ 5.0 µm Track-Etch Membrane (Whatman™, Cytiva, Freiburg im Breisgau, Germany) at room temperature. The properly dried samples were mounted on M4 cylinder SEM sample aluminum stub (Hitachi, Tokyo, Japan) using conductive carbon adhesive black tape and coated with a 10 nm thick gold layer. Deposition of IONPs on the surface of frustules was confirmed by EDAX and elemental mapping. The measurement was performed with an accelerating energy 30.0 kV using NSS Thermo Scientific.

### 4.4. Characterizations of Frustule Associated and External Biogenic IONPs

#### 4.4.1. UV-Vis Spectroscopy and Fourier Transform Infrared Spectroscopy

Control biomass, cleaned frustules (boiled with 30% H_2_O_2_ for 24 h and washed thrice with ddH_2_O) and IONPs loaded frustules of *P. trainorii* were sonicated with water using Hielscher UP100H ultrasonic processor (Teltow, Germany) for 20 min at 60% amplitude. After sonication, the experimental suspensions were centrifuged at 3000 rpm for 5 min and the supernatants were subjected to UV–Vis spectrophotometer DR 6000 (HACH–Lange) for optical measurements in the wavelength range of 200–900 nm. The UV-Vis spectra of orange-colored external counterpart was recorded before and after ultrafiltration in the 200–900 nm wavelength range by Nano Drop 2000c UV–Vis spectrophotometer (Thermo Fisher Scientific, Waltham, MA, USA).

FTIR measurements of control and IONPs loaded frustules of *P. trainorii* as well as extracellular IONPs (purified form) were carried out using ATR-FTIR (Bruker, Billerica, MA, USA). For FTIR measurement all samples were dried overnight at 50 °C and properly dried samples were subjected for IR measurements with wavenumber range of 4000–400 cm^−1^.

#### 4.4.2. Inductively Coupled Plasma Mass Spectrometry, Zeta Potential and X-ray Diffraction

After purification, the final concentration of external nanosuspension was quantified by inductively coupled plasma mass (ICP-MS) spectrometry (Agilent, Santa Clara, CA, USA). The zeta potential value of the synthesized spindle-shaped IONPs was measured by Malvern Zetasizer NanoZS (Malvern, UK) using Zeta cuvette DTS1070 (Malvern, UK). The analysis was carried out in the automatic selection mode of voltage and number of runs. Zeta potential measurements were performed in triplicate. The X-ray diffraction (XRD) spectra were recorded with an X’Pert Pro Analytical X-ray diffractometer (Philips, Würzburg, Germany) with Cu-Kα radiation (λ = 0.1541 nm, 40 kV, 30 mA); 1mL of dried sample on glass slide was scanned in the 2θ range between 5° and 100° with step sizes of 0.0167.

#### 4.4.3. TEM and EDAX

The frustule associated particles, extracted by sonication (20 min at 60% amplitude) following centrifugation at 3000 rpm for 5 min and ultrafiltered extracellular IONPs were dried on a carbon-coated copper grid (Sigma-Aldrich, St. Louis, MO, USA), and the size-shape analysis was carried out by a Hitachi STEM S5500 (Hitachi, Tokyo, Japan). The EDAX study was performed using the same grids and the same microscope (Hitachi STEM S5500 attached with EDAX) to understand the elemental composition and purity of the particles.

### 4.5. Photocatalytic Degradation of 4-Nitrophenol by IONPs Decorated Frustules

The degradation of 4-nitrophenol by IONPs loaded frustules were investigated by spectroscopy. To determine 4-nitrophenol degradation kinetics, 5 mg of IONPs doped frustules were suspended into 10 mL of 20 ppm 4-nitrophenol (pH 7.0) aqueous solution and the resultant suspension was stirred for 3 h under light at room temperature. The decolorization of 4-nitrophenol was measured by spectrophotometer at 400 nm at different reaction time points, namely 15, 30, 60, 75, 105, 120, 135, 150, 180 min. Before every measurement, the reaction mixture was centrifuged at 4000 rpm for 3 min to avoid the interference of IONPs loaded frustules. The experiment was performed in triplicates. A control set of 4-nitrophenol without IONPs loaded frustules was also maintained in the same reaction conditions.

The amount of nitrophenol degradation was calculated by this equation

% of 4-nitrophenol degradation = 100 × (C0 − C)/C0

where C0 is the initial and C is the concentration of nitrophenol after specific time of reaction [Concentration of 4-nitrophenol have been determined by calibration curve].

### 4.6. LDI-MS Analysis

Laser desorption ionization (LDI) analysis was performed using ultraFlextreme MALDI-TOF-MS apparatus (Bruker Daltonics, Bremen, Germany) equipped with a modified neodymium- doped yttrium aluminium garnet (Nd:YAG) laser operating at 355 nm and frequency 2 kHz. The plate (stainless steel, H17) was cut to pieces 2.5 × 7.5 cm, pre-cleaned using acetone, methanol and acetonitrile (10 min in ultrasonic bath) and inserted to MTP Slide-Adapter II (Bruker Daltonics, Bremen, Germany).

The LDI-MS spectra for characterization of IONPs were recorded in a reflectron positive mode in the range of *m*/*z* 60 to 3500 with the acceleration voltage of 5.5 kV, laser power 50%, global attenuator offset 30%. All spectra from a single spot were acquired by 5 laser shots with 500 laser frequency with 1.956 kV of reflector voltage. HCCA was used for external mass calibration. Blank area of metal plate was used as a negative control. All data were processed using using Flex Control (version 3.4, build 135) and Flex Analysis (version 3.4, build 50) software (Bruker Daltonics, Bremen, Germany). For characterization of IONPs, 1 µL of ultrafiltered extracellular IONPs was spotted to the plate.

The LDI-MS spectra for evaluation of LDI-MS efficiency of IONPs as nanomaterial assisting ionization/desorption were recorded in a reflectron positive mode in the range of *m*/*z* 60 to 1400, laser power 80%, global attenuator offset 30%, reflector voltage values as 26.63 and 13.59 kV, reflector voltage 1.940 kV with detector gain 2.51×. LDI-MS efficiency of IONPs was evaluated using standard solutions of glucose, alanine at concentration 1 mg/mL and mixture of triacylglycerols (50–125 µg/mL). Standard solutions of analytes were spotted using two approaches: (i) dried droplet, (ii) equal volumes (2.5 µL) of IONPs and analytes were pre-mixed and 1 µL of the mixture was spotted. External mass calibration was carried out using CsI_3_ solution and the procedure was as follows: equal volumes (2.5 µL) of 10 mg/mL of CsI_3_ in methanol and 20 mg/mL of DHB in methanol were premixed and 0.5 µL of the mixture was spotted. All spectra were collected using 2000 shots (500 shots × 4). Quadratic calibration model was used for external mass calibration.

## 5. Conclusions

In this study, a rapid, simple, eco-friendly technique has been proposed to synthesize IONPs in a cost-effective way. The selected strain, *P. trainorii* has been characterized as an efficient bioreagent for production of intra and extracellular IONPs within 3 days of reaction with 10 mM Fe^3+^ solution at room temperature. Luminescent property of the frustules/IONPs loaded frustules of *P. trainorii* can be exploited for various medical applications. IONPs loaded frustules showed efficiency in 94% of 4-nitrophenol degradation within 180 min of reaction. This catalytic activity of IONPs doped frustules can be used for nitrophenol removal from polluted water in future. The fabricated particles are all spindle-shaped and monodisperse in nature with well-marked hydrosphere surrounding the surface. The resultant structures can be utilized in various fields such as hyperthermia cancer therapy, bio-sensing SERS detection and electronic device designing, as well as in catalysis. The synthesized IONPs showed high positive zeta potential value and conferred high stability without any additional stabilizing agent. Bio-fabricated IONPs utilized as nanomaterial assisting ionization and desorption in LDI-MS showed intense signals for low molecular weight analytes at nano- and picomolar level (per spot). Therefore, the obtained results showed potential for the application of IONPs for the preparation of NALDI plates.

## Data Availability

Not applicable.
